# Resolution of central nervous system astrocytic and endothelial sources of CCL2 gene expression during evolving neuroinflammation

**DOI:** 10.1186/2045-8118-11-6

**Published:** 2014-03-04

**Authors:** Bandana Shrestha, Shujun Ge, Joel S Pachter

**Affiliations:** 1 Blood–brain Barrier Laboratory, Department of Cell Biology, University of Connecticut Health Center, 263 Farmington Avenue, Farmington, CT 06030, USA

**Keywords:** CCL2, MOG, Endothelial cells, Astrocytes, EAE, MS, CFA, PTX

## Abstract

**Background:**

The chemokine CCL2 is a critical mediator of neuroinflammation in diseases such as multiple sclerosis (MS) and its animal model, experimental autoimmune encephalomyelitis (EAE). CCL2 drives mononuclear cell infiltration into the central nervous system (CNS), alters expression and distribution of microvascular endothelial tight junction proteins, and disrupts the blood–brain and blood-spinal cord barriers. Immunohistochemistry has consistently revealed astrocytes to be a source of this chemokine during neuroinflammation, while providing less uniform evidence that CNS endothelial cells may also express CCL2. Moreover, the relative contributions of these cell types to the CNS pool of CCL2 during MS/EAE are unclear and the aim of this study was to investigate this further.

**Methods:**

CCL2 gene expression was determined by qRT-PCR in different populations of CNS cells at different times following EAE induced by immunization with MOG_35–55_ peptide and adjuvants, or after injection with adjuvants alone. CNS cells types were isolated by two different protocols: bulk isolation to yield crude microvascular and parenchymal fractions (containing astrocytes, other glia, and neurons), or laser capture microdissection (LCM) to acquire more precisely microvascular endothelial cells, astrocytes or other parenchymal cells.

**Results:**

Both CNS microvessel and parenchymal populations prepared by crude bulk isolation showed up-regulation of CCL2 mRNA following MOG immunization or injection of adjuvants alone. More exact dissection by LCM revealed microvascular endothelial cells and astrocytes to be the specific sources of CCL2 gene induction following MOG immunization, while only astrocytes showed elevated CCL2 mRNA in response to just adjuvants. Astrocytes displayed the greatest degree of stimulation of CCL2 gene expression following EAE induction.

**Conclusions:**

High-precision LCM affirmed both microvascular endothelial cells and astrocytes as the major CNS sources of CCL2 gene expression during EAE. Given the high accessibility of the CNS microvascular endothelium, endothelial-derived CCL2 could prove a viable target for therapeutic intervention in neuroinflammatory disease.

## Background

Numerous human and animal studies have highlighted the chemokine CCL2 (formerly known as MCP-1 [1]) as a critical mediator of the neuroinflammatory disease multiple sclerosis (MS) and its animal model experimental autoimmune encephalomyelitis (EAE) [2-4]. While long recognized for its chemotactic properties in guiding leukocyte migration, CCL2 has also been shown to destabilize tight junctions between microvascular endothelial cells that comprise the blood–brain and blood-spinal cord barriers [5-8]. This multifunctional status underscores CCL2’s value as a potential therapeutic target. However, for therapeutic measures to be most effective the sources of CCL2 in the central nervous system (CNS) have to be defined and the timing of CCL2 expression during neuroinflammation elaborated. While astrocytes are widely recognized in reviews as a major CNS source of CCL2 during MS and EAE, brain microvascular endothelial cells (BMEC) have received only limited acknowledgement for expressing this chemokine [9-11]. The scarce recognition of BMEC as a critical CCL2 source *in situ* may, in part, stem from the routine use of conventional immunohistochemistry to confirm expression. This technique may not be sufficiently sensitive to detect reliably perhaps smaller, vesicular quanta of endothelial CCL2 [12]. The use of adjuvants to induce EAE, e.g., Complete Freund’s Adjuvant (CFA) and pertussis toxin (PTX), can affect CCL2 expression in some endothelial cell types [13,14], and might further contribute to ambiguity of BMEC-derived CCL2 during neuroinflammatory disease. Previous reports have not examined the adjuvant issue. The status of BMEC CCL2 gene expression in neuroinflammation thus remains equivocal.

Herein, we report use of two highly-sensitive, qRT-PCR-based approaches to clarify the relative contributions of the parenchymal and vascular compartments to CNS CCL2 gene expression during the evolution of EAE induced by immunization with myelin oligodendrocyte glycoprotein (MOG) peptide_35–55_ along with adjuvants. One approach used a crude separation of CNS parenchymal and microvessel fractions, based on a common preparative method from homogenized CNS tissue. The other employed the laser capture microdissection (LCM), to more precisely retrieve separate BMECs, astrocytes and other parenchymal cell types. Additionally, we assessed CCL2 expression levels following MOG immunization as well as after injection of these adjuvants alone, to highlight the effects due to MOG immunoreactivity.

## Material and methods

### Animals

Female C57BL/6 mice, age 8–10 weeks were obtained from Charles River Laboratories, Inc. (Wilmington, MA, USA) and were euthanized by CO_2_ inhalation, following Animal Care and Use Guidelines of the University of Connecticut Health Center (Animal Welfare Assurance # A3471- 01). All the experimental procedures conducted have been approved under IACUC protocol #100346-1214.

### EAE induction

Mice were immunized with MOG_35–55_ peptide (MEVGWYRSPFSRVVHLYRNGK; W. M. Keck Biotechnology Resource Center, Yale University) as detailed previously [14]. Briefly, on day 0 (D0), female mice 7–9 weeks of age were injected subcutaneously with 300 μg of MOG peptide in Complete Freund’s Adjuvant (CFA) (DIFCO, Detroit, MI, USA) containing 1 mg/ml *Mycobacterium tuberculosis*. Mice were also injected intraperitoneally with 200 ng pertussis toxin (List Laboratories, Campbell CA, USA) in phosphate buffered saline (PBS) on D0 and D2 following MOG immunization. Mean clinical scores were calculated as the animals were monitored for clinical disease severity using the following: 0 = normal; 1 = tail limpness; 2 = limp tail and weak-ness of hind legs; 3 = limp tail and complete paralysis of hind legs; 4 = limp tail, complete hind leg and partial front leg paralysis; and 5 = death. The time-points selected for analysis, D9, D15 and D23, represent early EAE (score 0–0.5), acute EAE (score 2–2.5) and chronic EAE (score 2.5-3.5), respectively.

### Bulk isolation of parenchymal and microvessel fractions

Bulk isolations were prepared from each naïve, control and EAE group at all the time-points assessed, n = 3 mice for each group and time point. Separate parenchymal and microvessel fractions were obtained in bulk from freshly-dissected brain and spinal cord using a modification of previously described methods [15]. After removal of meninges and large blood vessels, one half of the brain and spinal cord tissue was homogenized in ice-cold PBS using a 7 mL Dounce tissue grinder (Kimble/Kontes, Vineland, NJ, USA). Brain and spinal cord tissue were combined for bulk isolation of parenchymal and microvessel fractions, in keeping with other reports detailing CNS CCL2 expression during EAE [16-18]. The other half spinal cord tissue was snap-frozen in dry ice-cooled 2-methylbutane (Acros; Geel, Belgium), and stored at − 80°C until used for LCM. The bulk homogenate was centrifuged at 400 × g for 15 min, and the resulting pellet resuspended in 18% (w/v) dextran (mw_r_ 60,000 – 90,000) and centrifuged at 4,500 × g for 10 min to sediment the crude “microvessel” fraction. The dextran supernatant and floating layer of myelinated axons were collected together to generate the crude “parenchymal” fraction and diluted in PBS; microvessels were resuspended in PBS and both fractions were washed twice by sedimentation at 720 × g for 10 min and rinsed in PBS. Microvessels were washed of blood cells by filtering through a 40 μm cell strainer (Becton Dickinson Labware, Franklin Lakes, IN, USA) and eluting with PBS. This procedure eliminated a significant fraction of the smaller capillaries, which passed through in the filtrate, while retaining the vast portion of larger venules on the filter [19]. The filter-bound microvessels were then solubilized with lysis buffer from the RNeasy Mini kit (QIAGEN, Valencia, CA, USA).

### Laser capture microdissection (LCM) of parenchyma, astrocytes and BMEC

Frozen spinal cords were embedded in cryomatrix compound (Thermo Fisher Scientific, Waltham, MA, USA), and 7 μm-thick frozen sections obtained. Immunohistochemstry-guided LCM was performed using a PixCell IIe laser capture microscope (Life Technologies Inc., Foster City, CA, USA), as previously described by this laboratory [20]. Significantly, this LCM approach has been shown to yield highly purified populations of microvascular endothelial cells and astrocytes, respectively. Tissue was captured from within the dorsolateral columns along the entire length of the spinal cord, as pathology in this EAE model proceeds up the CNS axis in a caudal-to-rostral direction [21], with lesions prominent in the spinal white matter. Briefly, anti-CD31 was used to label endothelial cells, along with alkaline phosphatase detection employing NBT-BCIP as chromogenic substrate. The endothelial cells of venules (10–50 μm in diameter) were specifically acquired [22] as these microvascular tributaries are the preferred sites of leukocyte extravasation [23], and were thus reasoned to have the highest CCL2 expression. This further allowed for a more equitable comparison with the bulk-isolated microvessels, which were enriched in venules. Anti-GFAP immunofluorescence was carried out to identify astrocytes in the same tissue sections. Areas of “other” parenchymal cells selected for LCM were those that did not stain with CD31 or GFAP, and thus contained neurons, oligodendrocytes and/or microglia. LCM tissue was solubilized in Cell Lysis Buffer® (Signosis; Sunnyvale, CA, USA).

### RNA isolation, cDNA synthesis and qRT-PCR

Total RNA was isolated from bulk microvessel and parenchymal fractions using the RNeasy Mini kit (QIAGEN), and treated with Turbo DNAse (Ambion, Austin, TX, USA). cDNA was generated using SuperScript III (Invitrogen, Carlsbad, CA, USA) and relative CCL2 RNA level determined by qRT-PCR using SYBR green (AB Applied Biosystems, Foster City, CA, USA) as described previously [24]. LCM tissue was subjected to DNAse treatment using Turbo DNAse followed by reverse transcription, using SuperScript III. LCM-derived cDNA was then pre-amplified using TaqMan PreAmp Master Mix and a PreAmp Pool containing all primers for detection by the Mouse Immune Panel TaqMan Low Density Array (TLDA; Life Technologies Corp., Foster City, CA, USA) and probed for CCL2 by qRT-PCR using a single-plex gene expression assay [14]. An ABI 7900HT fast real-time PCR System (Life Technologies Corp.) was used to detect amplicon amount for both bulk and LCM preparations. RPL-19 was used as a reference for relative CCL2 gene expression for bulk isolation as detailed previously [24] and GAPDH for LCM preparation [14], both genes having been shown to be largely invariant during EAE. Relative quantitation was performed using the 2-[Δ] [Δ] Ct method of Fleige *et al.*[25]. Results were analyzed using a one-way non-parametric Kruskal-Wallis test followed by Dunn’s post-test analysis using GraphPad Prism 5 (GraphPad, La Jolla, CA, USA). Results were considered significant at *P* ≤ 0.05.

## Results and discussion

**Figure 1:**
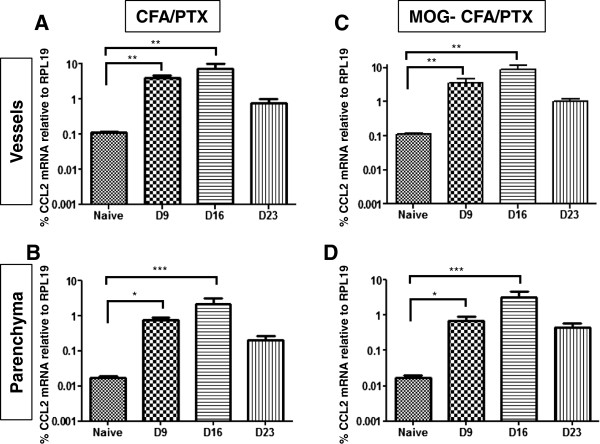
**CCL2 gene expression in CNS microvessel and parenchymal fractions obtained by bulk preparation.** Separate, crude microvessel **(A, C)** and parenchymal **(B, D)** fractions were prepared by bulk isolation from combined brain and spinal cord tissue of mice injected with CFA/PTX **(A, B)** or immunized with MOG-CFA/PTX **(C, D)**. Analyses were performed at D9, D16 and D23 following beginning of either injection regime, and relative CCL2 mRNA levels are plotted on a log scale. Comparisons were made to naïve mice. *P ≤ 0.05; **P ≤ 0.01; and ***P ≤ 0.001. Data has been presented as mean +/− SEM. For each experiment, bulk isolations were performed from three individual mice from each naïve, control (CFA/PTX) and EAE (MOG-CFA/PTX) group, and experiments were conducted twice.

CCL2 expression levels were first evaluated in separate parenchymal and microvessel fractions prepared by bulk isolation from brain and spinal cord tissue of either MOG-CFA/PTX-treated (EAE) or CFA/PTX-treated (Control) mice. Analysis was performed at different days after initial injection, which represented onset (D9), peak (D16) and chronic (D23) phases of clinical EAE disease (Figure 1). As determined by qRT-PCR, relative expression of CCL2 by microvessels was significantly higher in both treatment groups (CFA/PTX and MOG-CFA/PTX) compared to naïve animals at D9 and D16, with a return to naïve level by D23 (Figure 1A, C). The parenchymal fraction showed a similar trend of significantly increased CCL2 expression in both treatment groups compared to naïve animals at D9 and D16 (Figure 1B, D).

**Figure 2:**
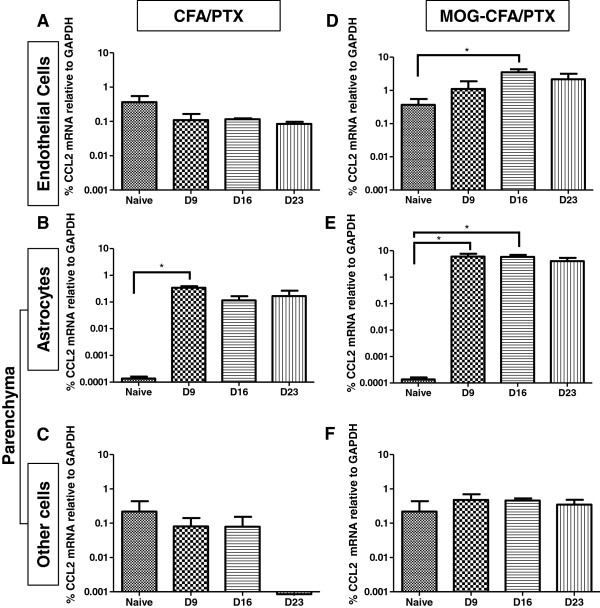
**CCL2 gene expression in CNS microvascular endothelial cells, astrocytes and other parenchymal cells isolated by LCM.** Endothelial cells **(A, D)**, astrocytes **(B, E)**, and other parenchymal cells **(C, F)** were separately acquired by LCM from spinal cord tissue of mice injected with CFA/PTX **(A-C)** or immunized with MOG-CFA/PTX **(D-F)**. Analyses were performed at D9, D16 and D23 following either injection regime and relative CCL2 mRNA levels are plotted on a log scale. Comparisons were made to naïve mice. *P ≤ 0.05; **P ≤ 0.01; and ***P ≤ 0.001. Data has been presented as mean +/− SEM. For each experiment, LCM was performed on tissue from three individual mice from each naïve, control (CFA/PTX) and EAE (MOG-CFA/PTX) group.

Next, we performed a time-course analysis with CNS tissue that was more precisely dissected into endothelial cells (CD31), astrocytes (GFAP) and “other” parenchymal cells (selected areas devoid of CD31 and GFAP immunostaining) by LCM. As was the case with bulk preparation of microvessels, endothelial cells showed elevated CCL2 expression in MOG-CFA/PTX-treated mice at D16 compared to naïve mice (Figure 2D). However, corresponding endothelial samples from CFA/PTX-treated mice did not show a similar increase (Figure 2A). Astrocytes exhibited significantly elevated CCL2 expression at both D9 and D16 in the MOG-CFA/PTX-treated group (Figure 2E), and a significant increase at D9 in the CFA/PTX-treated cohort (Figure 2B). In contrast to that observed with the bulk parenchymal preparations, other parenchymal cells isolated by LCM failed to display an increase in CCL2 expression either in MOG-CFA/PTX-treated (Figure 2F) or CFA/PTX-treated (Figure 2C) mice compared to naïve mice at any time-point.

These findings underscore several points. Expression of CCL2 was significantly induced in both astrocyte and endothelial cell populations in the MOG-induced EAE paradigm. The fact that CCL2 mRNA level appeared higher in endothelial cells than in astrocytes in naïve mice may reflect the capture of some circulating leukocytes along with endothelial cells, as the mice were not perfused prior to LCM. Interestingly, while CFA/PTX injection resulted in elevated CCL2 expression in isolated microvessels, it did not do so in LCM-acquired endothelial cells. This suggests that sources other than endothelial cells contributed to the altered CCL2 mRNA level in the bulk microvessel fraction. As astrocytes lie in close proximity to endothelial cells within the neurovascular unit (NVU) [26,27] and can contaminate microvessel preparations [28,29], *a priori* these particular glial cells may be a significant source of CCL2 mRNA detected in bulk-isolated microvessels. This caveat reinforces LCM as a critical technology to resolve more effectively sources of gene expression *in situ*.

Our LCM results further suggest astrocytes are the preeminent parenchymal sources of induced CCL2 gene expression during EAE, since the other parenchymal cells acquired by this technique showed no significant stimulation of CCL2 mRNA. As increased immunostaining of CCL2 in microglia has been reported in EAE [30], this may represent up-regulation at the protein rather than the RNA level. It may also be that the percentage contribution of microglia in the other parenchymal cells acquired by LCM, was not high enough to show overall elevation of CCL2 gene expression in these samples from diseased mice. CCL2 mRNA detected in other parenchymal cells from naïve mice could possibly also reflect a low level of constitutive CCL2 expression by neurons [31].

Lastly, CFA/PTX alone can stimulate CCL2 expression in astrocytes (Figure 2B), possibly providing a priming function for supernumerary stimulation due to MOG effects (Figure 2E). This is consistent with reports that peripheral inflammation induced by CFA or Mycobacterium resulted in CNS glial activation [32,33]. Previous results from this laboratory indicated CFA/PTX injection also up-regulated expression of CCL2 by choroid plexus capillary endothelial cells. Thus, it is imperative that any measure of gene expression following the typical MOG immunization protocol be compared to effects seen after injection of CFA/PTX alone, to discern what gene changes are associated specifically with MOG-induced pathogenesis.

These results reinforce the CNS microvascular endothelium as a significant source of CCL2 [9,10,12], as well as spotlight LCM as a critical tool for the selective enrichment of vascular or other CNS cell types for gene expression studies [14,20,22]. Moreover, given the microvascular endothelium is highly accessible from the circulation as compared to the astrocyte population which lies behind the BBB and BSCB, endothelial-derived CCL2 may be a “druggable” target in neuroinflammatory disease [24,34].

## Abbreviations

BBB: Blood brain barrier; BMEC: Brain microvascular endothelial cells; CFA: Complete Freund’s adjuvant; CNS: Central nervous aystem; EAE: Experimental autoimmune encephalomyelitis; LCM: Laser capture microdissection; MOG: Myelin oligodendrocyte glycoprotein; MS: Multiple sclerosis; PTX: Pertussis toxin.

## Competing interests

The authors declare that they have no competing interests.

## Authors’ contributions

BS aided in the design and implementation of the experiments, carried out bulk isolation of CNS tissues, performed immuno-LCM for resolution of CNS CCL2 sources and contributed to the writing and editing of the manuscript. SG assisted with the immunization and bulk isolation procedures. JP designed the experiments, wrote the manuscript and provided oversight for the project. All authors have read and approved the final version of the manuscript.
